# On the Para/Ortho Reactivity of Isocyanate Groups during the Carbamation of Cellulose Nanocrystals Using 2,4-Toluene Diisocyanate

**DOI:** 10.3390/polym11071164

**Published:** 2019-07-08

**Authors:** Hatem Abushammala

**Affiliations:** Fraunhofer Institute for Wood Research (WKI), Bienroder Weg 54E, 38108 Braunschweig, Germany; hatem.abushammala@wki.fraunhofer.de

**Keywords:** cellulose, nanocellulose, toluene diisocyanate, isocyanate, carbamation

## Abstract

2,4-toluene diisocyanate (TDI) has been commonly used to bind molecules and polymers onto the surface of cellulose nanocrystals (CNCs). Such a process usually involves two steps: (1) the more reactive para-isocyanates (p-NCOs) of TDI are reacted with the surface hydroxyl groups of CNCs then (2) the ortho-isocyanates (o-NCOs) are reacted with certain desired molecules. During the first reaction, an ideal para/ortho selectivity could be impossible to achieve, as o-NCOs are not fully unreactive. Therefore, there is a need to better understand the reaction between CNCs and TDI towards a maximum para/ortho selectivity. For that goal, CNCs were reacted with TDI under varying temperatures (35–75 °C) and TDI/CNCs molar ratios (1–5). The amount of the reacted TDI was estimated using elemental analysis while the free o-NCO groups were quantified following the hydrolysis method of Abushammala. The results showed that temperature had a negative impact on para/ortho selectivity while TDI/CNCs molar ratio improved it. A maximum selectivity of 93% was achieved using a temperature of 35 °C and a molar ratio of 3. This is a three-fold improvement to that using the traditional reaction conditions (75 °C and molar ratio of 1).

## 1. Introduction

**Figure 1:**
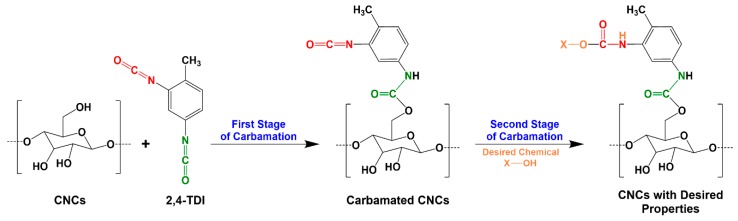
The functionalization of cellulose nanocrystals (CNCs) with a desired chemical using 2,4-toluene diisocyanate (TDI) as a linker [23].

Cellulose nanocrystals (CNCs) are rod-like nanoparticles with a thickness of 3–10 nm and a length of few hundreds of nanometers [1]. They can be extracted from cellulose or directly from wood using a variety of procedures and reagents including sulfuric acid and ionic liquids [2,3,4,5]. To widen the range of their applications, CNCs lend themselves to many kinds of surface modifications such as acetylation and oxidation. Functional polymers and chemicals have been also grafted onto the CNC surface [6,7,8,9,10]. These modifications do not only functionalize the CNCs, but can also improve their properties in general and their miscibility in dispersions and matrices towards the fabrication of nanocomposites with improved interfacial adhesion [11,12]. During modification, a linker such as 2,4-toluene diisocyanate (TDI) is sometimes needed to bind certain chemical functionalities onto a CNC surface [13,14,15]. TDI is mostly used for the production of polyurethanes [16,17,18] and it is known for the difference in the reactivity of its two isocyanate groups; ortho isocyanate (o-NCO) is 5–10 times less reactive than the para one (p-NCO) due to the steric hindrance imposed by the methyl group [19,20]. This characteristic made TDI very interesting to graft a variety of chemicals and polymers onto the surface of CNCs [8,14,15,21]. In such a process, TDI reacts with the hydroxyl groups of the CNC surface through the more reactive p-NCO (first stage) followed by reacting the less reactive o-NCO with a desired chemical/polymer (second stage) (Figure 1). In reality, this reaction is difficult to control as o-NCOs are not fully unreactive during the first stage [22], which means that a fraction of the TDI on the CNC surface has both o-NCO and p-NCO reacted during the first stage and becomes therefore dysfunctional for the later grafting. This possibly explains the inability of some studies to graft certain functional chemicals onto a CNC surface in amounts equivalent to the amount of TDI placed earlier on that surface as not all the TDI molecules thereon have free isocyanates [9]. Therefore, a deeper understanding and a thorough optimization of the carbamation reaction of TDI with CNCs are needed.

In a previous report, the author has developed a simple method for quantifying the isocyanates (o-NCO) available on the CNC surface upon TDI-based carbamation [23]. It relies on the pH increase upon hydrolyzing the free isocyanates of the carbamated CNCs using HCl-acidified dimethyl sulfoxide (DMSO) as a hydrolysis medium. This method made it possible to determine the fraction of TDI on the CNC surface that has its o-NCO free and that has both of its isocyanates reacted. The method showed that the traditional carbamation conditions should be more thoroughly optimized as only one third of the TDIs, on the CNC surface, have free o-NCOs that are available for a following grafting. Towards optimum carbamation, this paper investigates the impact of temperature and TDI/CNCs molar ratio on the amount of TDI (DS_TDI_) and free isocyanates (DS_NCO_) on the CNC surface upon carbamation and, consequently, on the carbamation efficiency (DS_NCO_/DS_TDI_).

## 2. Materials and Methods

### 2.1. Materials

CNC suspension (solid content of 10.4% (*w*/*w*)) was purchased from the University of Maine (Orono, ME, USA), which was prepared using the sulfuric acid method. Acetone (≥99%), toluene (≥99%), triethylamine (TEA) (≥99%), DMSO (≥99%), chloroform (≥99%), and hydrochloric acid (37%) were purchased from VWR (Darmstadt, Germany). 2,4-toluene diisocyanate (≥98%) was purchased from TCI Chemicals (Eschborn, Germany) and was stored in sealed bottles in the fridge. Toluene, TEA, and DMSO were stored over A4 molecular sieve, while acetone was stored under A3 molecular sieve. Both molecular sieves were purchased from Carl Roth (Karlsruhe, Germany) and regenerated before use.

### 2.2. TDI-Carbamation of Cellulose Nanocrystals

The CNCs were carbamated following the method of Habibi and Dufresne after small modifications [13]. A total of 9.6 g of 10.4% CNC suspension, which is an equivalent to 1.0 g of dried CNCs (6.2 mmol), was solvent-exchanged from water to anhydrous toluene using the following washing/precipitation protocol. The CNCs were washed and precipitated three times with anhydrous acetone then twice with anhydrous toluene. The precipitation was performed using a Sigma 3-16P centrifuge (g-force of 4472, 5000 rpm for 30 min) (Sigma Laborzentrifugen, Osterode am Harz, Germany). After the final washing with toluene, the precipitated CNCs were transferred using a certain volume of anhydrous toluene to a 100 mL round-bottom flask. The amount of toluene varied to keep the final reaction volume constant (ca. 52 mL) (Table 1). Certain amounts of 2,4-TDI and triethylamine (TEA) as catalyst were transferred to the reaction flask (Table 1). The reaction proceeded at a certain temperature in a moisture-free environment (nitrogen). After 24 h, the reaction mixture was centrifuged to collect the carbamated CNCs, which were then washed once with anhydrous toluene and twice with anhydrous DMSO. The collected CNCs were finally transferred to the acidified DMSO for hydrolysis and quantification of free isocyanates. To assure reproducibility, the reaction was performed in duplicate. For characterization, the carbamated CNCs were washed with anhydrous toluene (three times) instead of DMSO and oven dried overnight under vacuum at 50 °C.

### 2.3. Determination of the Degree of Substitution of TDI (DS_TDI_)

The elemental composition of the original and carbamated CNCs was determined using the scanning electron microscope ZEISS GeminiSEM Crossbeam 340 (ZEISS, Oberkochen, Germany), which was equipped with the energy dispersive X-ray (EDX) detector X-MaxN (Oxford Instruments, Abingdon, UK). The powdered samples were pressed using a mold to obtain discs (diameter: 1 cm) of smooth surfaces. The elemental composition of the discs was determined using a voltage of 10 kV. DS_TDI_ was then estimated based on the increase in the nitrogen/carbon molar ratio (R) (see the Supplementary Materials):

(1) $$DS_{TDI}=\frac{mmol\;TDI}{mmol\;CNC\;Hydroxyls}=168,796.6\;R^{4}-27,737.8\;R^{3}+2,270.4\;R^{2}+36.7\;R+0.1.$$

### 2.4. Determination of the Degree of Substitution of Free Isocyanates (DS_NCO_)

The degree of substitution of isocyanates (DS_NCO_) was determined following the method of Abushammala [23] (see the Supplementary Materials). 

### 2.5. Morphological Characterization using X-Ray Diffraction (XRD)

The crystalline structure of the original and carbamated CNCs was analyzed using a Bruker AXS D8 X-ray diffractometer (Bruker, Billerica, MA, USA) using a CuKα1 radiation, which operated at 40 kV and 40 mA. The intensities were collected at 2θ from 5° to 40° using the software Diffrac Plus X-Ray Diffraction (XRD) Commander. Peak deconvolution was performed using the software PeakFit (version 4.12) to determine the areas of the characteristic peaks of the crystalline diffractions at 2θ of 14.8°, 16.3°, 20.4°, 22.4°, and 34.5° and the amorphous diffraction at 2θ of 18°. The crystallinity (Cr) was then calculated [24]:

(2) $$Cr=\frac{Total\;Area\;of\;Crystalline\;Diffraction\;Peaks}{Total\;Area\;of\;Crystalline\;and\;Amorphous\;Diffraction\;Peaks}*100\%.$$

### 2.6. Morphological Characterization using Atomic Force Microscopy (AFM)

A drop of a diluted suspension (10^−4^% *w*/*w*) of the CNCs before and after carbamation was deposited on a fresh mica surface then kept to air-dry overnight. The original CNC suspension was in water while the carbamated CNCs were suspended in chloroform. The surface was imaged using the atomic force microscope Agilent 5500 (Keysight Technologies, Santa Rosa, CA, USA). The height images were obtained in the tapping mode using the silicon tips PPP-NCH (Wetzler, Nanoandmore, Germany), which had a spring constant of ca. 50 N.m^−1^ and a resonance frequency of ca. 350 kHz. The thickness of the CNCs was determined based on a sample size of 100 particles using the Gwyddion software (version 2.53, Czech Metrology Institute, Brno, Czech).

## 3. Results and Discussion

### 3.1. Estimation of the Percentage of Hydroxyl Groups on the CNC Surface

To monitor the extent of the carbamation reaction, it is important to estimate the amount of reactive hydroxyl groups on the CNC surface as the reaction supposedly takes place on there at first, before targeting the inside. The CNCs used in this work have a thickness of 7.0 ± 1.6 nm as measured using AFM. Based on the crystallographic results of Nishiyama, these CNCs are made of 14 × 14 cellulose chains, out of which 52 are on the surface [25]. These 52 chains have 168 hydroxyls groups per a cross section unit (cellubiose unit) compared to a total 1176. Therefore, these CNCs have 14.3% of their hydroxyl groups on the surface. However, many studies have shown that C3 hydroxyls (equivalent to a DS of 4.8%) are unreactive due to steric and inductive effects and due to their involvement in hydrogen bonding [26,27]. Moreover, a significant fraction of the C6 hydroxyls is sulfonated by sulfuric acid as a side-reaction during the production of the CNCs [28,29,30]. Using elemental analysis, 42% of the C6 hydroxyls were sulfonated (equivalent to a DS of 2.0%). Therefore, the maximum possible degree of substitution for these CNCs is 7.5%. In addition to surface hydroxyls, a significant amount of hydroxyls is available in the amorphous regions of the CNCs in both the longitudinal and transverse directions. These hydroxyls, however, are less accessible than the surface hydroxyls due to the use of toluene as a non-swelling solvent for carbamation [31]. Indeed, the hydroxyls in the crystalline regions are the least accessible.

### 3.2. Impact of Temperature and TDI/CNCs Molar Ratio on Carbamation Efficiency

When TDI reacts with a hydroxyl group on the CNC surface, it is assumed that only its para-NCO is reactive while o-NCO stays free and does not get involved in any reaction. This assumption is based on the 5–10 time difference in p-NCO/o-NCO reactivity [19,20]. This assumption is indeed invalid as a certain fraction of o-NCO does react reducing the efficiency of carbamation and the amount of desired molecules/functionalities, which is possible to graft on the CNC surface at a later stage [23]. Therefore, this work aims at optimizing the carbamation conditions towards a maximum para/ortho selectivity. In a previous work by the author, a method was developed to quantify the free isocyanates available on the CNC surface (degree of substitution of isocyanates, DS_NCO_) upon TDI-based carbamation. The method relies on the pH increase upon a complete hydrolysis of isocyanates to amine groups using HCl-acidified DMSO. On the other hand, elemental analysis can be used to estimate the amount of the reacted TDI on the CNC surface (degree of carbamation, DS_TDI_) by following the increase in nitrogen/carbon molar ratio (see Supplementary Materials). Combining both methods, it is possible to determine the fraction of TDI on the CNC surface that has a free isocyanate (DS_NCO_/DS_TDI_) and, therefore, assess the efficiency of carbamation.

A typical TDI carbamation of CNCs involves toluene as a non-swelling solvent for cellulose to allow the carbamation to take place mainly on the CNC surface (heterogeneous conditions) [31,32], and triethylamine (TEA) as one of the most commonly used catalysts for carbamation [33]. Therefore, in addition to time and temperature, the amounts of TDI, CNCs, toluene, and TEA are important process parameters to investigate. For simplicity, TDI/TEA ratio, reaction volume, and time were kept constant. Time was excluded because TDI-carbamation of CNCs is a slow process. Usually, 24 h are needed for the reaction to progress significantly. The reaction volume was kept constant (ca. 52 mL) by slightly changing the volume of toluene (Table 1). Therefore, the nature of CNCs carbamation using TDI was studied by only varying the carbamation temperature and TDI/CNCs molar ratio (Table 1). The range of temperature was selected to cover lower temperatures than that used in the traditional carbamation procedure (75 °C and molar ratio of 1). Higher molar ratios were explored only with low temperatures to prevent the reaction from happening under homogeneous conditions (CNC dissolution).

**Figure 2:**
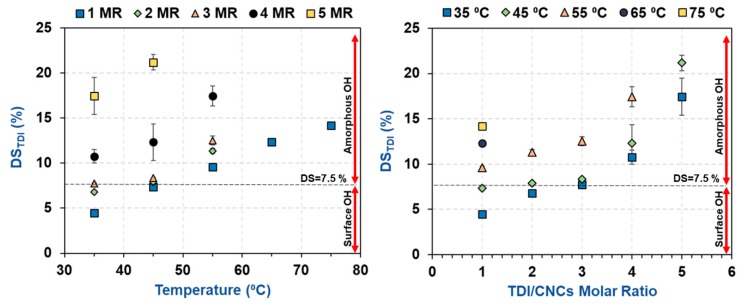
The impact of carbamation temperature and TDI/CNCs molar ratio on the amount of TDI reacted with CNCs, i.e., degree of TDI substitution (DS_TDI_).

The results showed that the degree of carbamation (DS_TDI_) increased with increasing temperature and TDI/CNCs molar ratio (Table 2 and Figure 2) as both factors are expected to increase the kinetics of carbamation and the accessibility of TDI to the CNC hydroxyls. It was however interesting that, at 35 and 45 °C and using a molar ratio of up to 3, a plateau can be seen around a DS of 7.7%. This value is in agreement with the percentage of hydroxyls available on the CNC surface (7.5%) as estimated earlier using Nishiyama’s crystallographic results. This implies that at low temperatures and molar ratios, carbamation takes place mainly on the CNC surface. Using higher temperatures and/or molar ratios led to higher DS_TDI_ values, possibly due to the reaction of TDI with the amorphous hydroxyls. At such severe conditions, the hydroxyl groups inside the crystals might also become reactive. This, if happened, would have a severe effect on the crystallinity of the CNCs.

**Figure 3:**
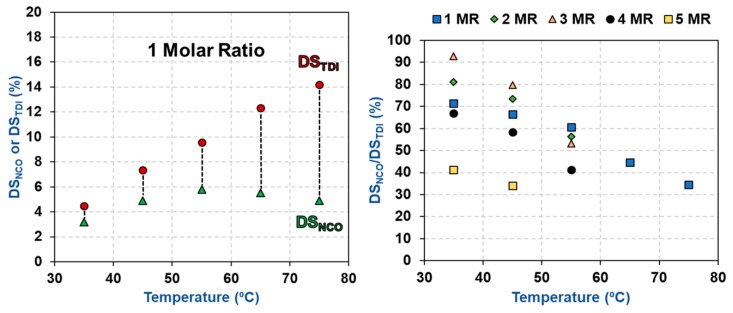
The impact of carbamation temperature on DS_NCO_, DS_TDI_, and carbamation efficiency (DS_NCO_/DS_TDI_).

The degree of substitution of isocyanates (DS_NCO_) was then determined for the carbamated CNCs using the hydrolysis method of Abushammala. In an ideal situation, the amount of isocyanates (DS_NCO_) and TDI (DS_TDI_) should be identical, as only the p-NCOs of TDI should react with the CNC hydroxyls while the o-NCOs must be fully unreactive. This is unfortunately not the case as the carbamation temperature had a negative impact on the amount of free isocyanates (Table 2 and Figure 3). Using the traditionally-used carbamation conditions (75 °C and molar ratio of 1) only one third of the TDIs (DS_NCO_/DS_TDI_ = 4.9%/14.2% = 34%), which reacted with the CNCs, had a free isocyanate. This means that two thirds of the reacted TDI had both of their isocyanates (ortho and para) reacted making these two thirds of TDI dysfunctional for any following grafting of desired functionalities. Using 35 °C and molar ratio of 1, the reaction efficiency improved significantly, as more than two thirds of the reacted TDIs had a free NCO (DS_NCO_/DS_TDI_ = 3.2%/4.5% = 72%). This behavior can be understood when considering the kinetics parameters of carbamation. o-NCO has a significantly higher activation energy of carbamation (ca. 20 kcal/mol) compared to p-NCO (ca. 10 kcal/mol). However, high temperatures make it possible to overcome both energy barriers and allow carbamation to take place by both isocyanates at comparable rates [34].

**Figure 4:**
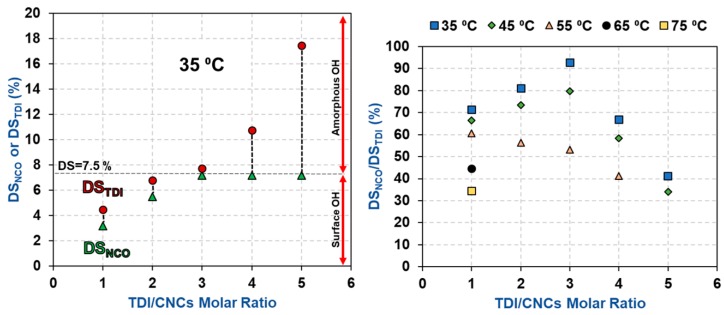
The impact of TDI/CNCs molar ratio on DS_NCO_, DS_TDI_, and carbamation efficiency (DS_NCO_/DS_TDI_).

The impact of TDI/CNCs molar ratio on carbamation efficiency was more complicated as it was dependent on the extent of carbamation (Table 2 and Figure 4). When carbamation took place mainly on the CNC surface (using a temperature of 35 or 45 °C and molar ratio of up to 3), increasing the molar ratio improved the reaction efficiency. For instance, using 35 °C and molar ratio of 3, 93% of the reacted TDI had a free isocyanate (DS_NCO_/DS_TDI_ = 7.2%/7.7%), which is the maximum selectivity obtained. It is a significant improvement when compared to a DS_NCO_/DS_TDI_ of 72% using 35 °C and 1 molar ratio. It seems that increasing the amount of TDI in the reaction mixture lowered the chances of o-NCO to react with the CNC hydroxyls by increasing the number of p-NCOs per a hydroxyl group. On the other hand, when carbamation was more severe (high temperature and/or high TDI/CNCs molar ratio) and progressed further in the amorphous areas and wherever possible inside the crystallites, the molar ratio had a negative impact on carbamation efficiency (DS_NCO_/DS_TDI_ of 43% using 35 °C and a molar ratio of 5). Despite the reaction of further amounts of TDI with CNCs, DS_NCO_ surprisingly stayed constant (7.2%). This implies that the additional TDI, which reacted with the amorphous regions of the CNCs, had both of their isocyanates reacted. This behavior is clearer for the CNCs carbamated at 55 °C, where the reaction took place in the amorphous areas even at a low TDI/CNCs molar ratio of 1. Increasing the molar ratio in this case always had a negative impact on carbamation efficiency in contrast to when carbamation took place at 35 and 45 °C. This could be explained by considering the relatively rigid structure of TDI and the high accessibility of hydroxyl groups in the amorphous areas [35,36]. When a TDI molecule reacts through its p-NCO with a hydroxyl group on the CNC surface, it becomes difficult for the o-NCO to react with a neighboring hydroxyl due to geometrical and structural hindrances. The scenario, however, becomes easier when the reaction takes place in the amorphous regions of CNCs as the hydroxyl groups there are more accessible and more reactive [37,38]. In conclusion, the optimum carbamation conditions are the ones, by which carbamation takes place mainly on the CNC surface. Using 35 °C and molar ratio of 3, all the hydroxyls on the CNC surface reacted with TDI (DS_TDI_ = 7.7%) and 93% of these TDIs had a free isocyanate (DS_NCO_/DS_TDI_ = 7.2%/7.7% = 93%).

**Figure 5:**
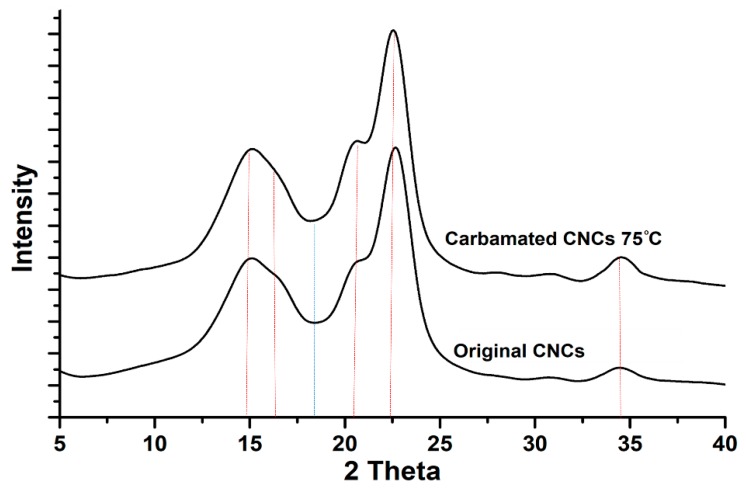
The crystalline structure of the CNCs before and after carbamation at 75 °C using a TDI/CNCs molar ratio of 1.

**Figure 6:**
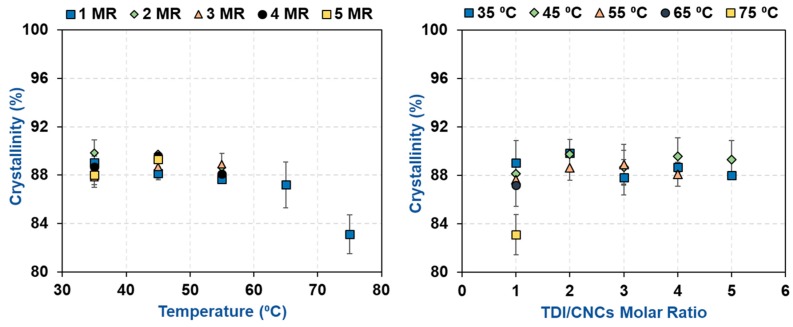
The impact of carbamation temperature and TDI/CNCs molar ratio on the crystallinity of CNCs upon carbamation.

For the carbamated CNCs with a DS_TDI_ of more than 7.5% (high temperature and/or high TDI/CNCs molar ratio), the carbamation reaction took place not only on the surface but also in the amorphous areas and possibly inside the crystallites as well. To shed more light on that, the crystalline structure of the CNCs before and after carbamation was studied. The diffractograms of all carbamated CNCs, in addition to the original CNCs, showed the native Cellulose I crystalline structure with five diffraction peaks at 2θ of 14.8°, 16.3°, 20.4°, 22.4°, and 34.5° (Figure 5). No Cellulose II crystalline structure was observed at 2θ of 12° [39]. Upon peak deconvolution, the crystallinity of the samples was determined using the peak areas of the crystalline and amorphous diffractions (Figure 6). In general, the crystallinity of the CNCs after carbamation did not change significantly when compared to the crystallinity of the original CNCs (90.2%). TDI/CNCs molar ratio showed no significant impact on crystallinity whatsoever. This means that higher molar ratios of 4 and 5 could only facilitate the reaction of TDI with the hydroxyl groups of the amorphous regions and imperfections of CNCs without affecting the crystallites. Temperature showed no significant impact on crystallinity at low reaction temperatures (up to 55 °C). Temperatures higher than 55 °C led to a decrease in crystallinity to a minimum of 83.1% using 75 °C indicating the reaction of TDI with hydroxyl groups within the crystallites. This drop in crystallinity recommends the use of low temperatures for carbamation to keep the reaction happening mainly on the CNC surface, where also the para/ortho selectivity is the highest as shown earlier.

### 3.3. Possible Side Reactions during TDI-Based Carbamation

**Figure 7:**
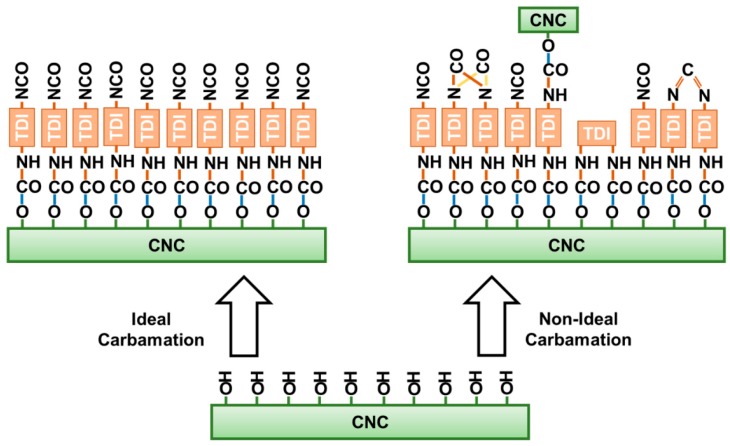
Possible side reactions of the isocyanates of TDI during CNC carbamation.

A side from the ideal carbamation of CNCs using TDI, where the only possible reaction is that between the hydroxyls of CNCs and the p-NCOs of TDI, some side reactions can also take place (Figure 7). These side reactions would decrease the amount of free isocyanates on the CNC surface. TDI, itself, in the presence of a catalyst and heat can self-polymerize through its isocyanates [40,41]. This may happen to both the TDI molecules in the reaction mixture and those on the CNC surface. Such side reaction could possibly lead to the formation of the dimers: uretidinedione and carbodiimide. It is unlikely that trimers and higher oligomers would form on the CNC surface due to steric and structural hindrances [42]. Despite of the possibility of self-polymerization to happen, it does not seem as a major side reaction as the amount of dimers is not significant enough to be detected using spectroscopic methods such as Fourier-transform infrared spectroscopy.

**Figure 8:**
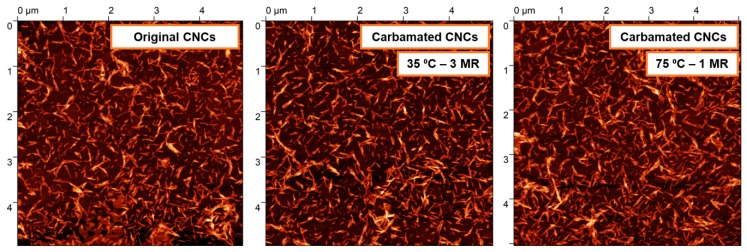
AFM topography images of CNCs before and after carbamation indicating no significant agglomeration or change in morphology. MR stands for TDI/CNCs molar ratio.

The side-reaction, which is expected to mostly happen, is the reaction of both isocyanates of TDI with CNC hydroxyls leading to crosslinking. The isocyanates of a TDI molecule may react with hydroxyl groups from the same CNC particle or with hydroxyl groups from two CNCs. When it takes place in the same CNC, it is expected to be more dominant in the amorphous areas as they offer the rigid TDI molecules the needed geometrical accessibility to hydroxyl groups for both isocyanates to react. Moreover, the reactivity of hydroxyl groups including the C3 hydroxyls is strongly higher in the amorphous regions as the hydrogen bonding is significantly weaker there [37,38]. This was implied by the results earlier as increasing the TDI/CNCs molar ratio to more than 3 pushed the carbamation reaction to happen on the CNC surface and in the inside but with no increase in the free isocyanate content whatsoever. This implies that all of the TDI molecules reacted in the amorphous regions of the CNCs had both of their isocyanates reacted. Since it would mainly happen in the amorphous regions, this kind of crosslinking is not expected to change the CNC morphology. In contrary, a crosslinking between two CNCs would push them to agglomerate or stack as they become “glued” to each other. This kind of CNC-to-CNC crosslinking seems to rarely happen as no significant agglomeration was observed using AFM for the CNCs carbamated at 35 °C and molar ratio of 3 (DS_NCO_/DS_TDI_ = 93%) and 75 °C and molar ratio of 1 (DS_NCO_/DS_TDI_ = 34%) when compared to the original CNCs (Figure 8). The CNC thickness for both carbamated samples, 7.8 ± 1.7 nm and 8.0 ± 1.0 nm respectively, is only slightly higher than that for the original CNCs (7.0 ± 1.6 nm) indicating no CNC stacking. This slight increase in CNC thickness might be due to the shell of TDI molecules, which was built up around the CNC particles upon carbamation.

To summarize, the carbamation conditions had a strong impact on its efficiency. They determined the extent of carbamation and the fraction of TDIs with free ortho isocyanates. The ortho isocyanates, which were not free upon carbamation, may have undergone a crosslinking reaction with a neighboring hydroxyl or with a hydroxyl from another CNC particle, or have undergone a dimerization reaction with other surrounding isocyanates. These side reactions seem to be suppressed at low carbamation temperatures. For instance, at a low temperature of 35 °C (and TDI/CNCs molar ratio of 3), the surface of CNCs was fully covered with TDI, 93% of which had a free isocyanate. The role of TDI/CNCs molar ratio in these side reactions is more complicated. It is expected that increasing the molar ratio would reduce crosslinking since it makes it more competitive for the o-NCOs to react with the CNC hydroxyl groups, as more p-NCOs are present in the reaction mixture. At the same time, more TDI in the reaction mixture should increase the chances of isocyanates, in general, to self-polymerize. The results, however, showed that increasing the molar ratio improved carbamation efficiency. This confirms that crosslinking, not self-polymerization, is the dominant side-reaction during CNC carbamation.

## 4. Conclusions

The carbamation of CNCs using TDI was optimized by investigating the impact of temperature and TDI/CNCs molar ratio on the amount of TDI reacted with CNCs (DS_TDI_) and the amount of free isocyanates available on the CNC surface (DS_NCO_). It was evident that temperature had a negative impact on carbamation efficiency by allowing the o-NCOs of TDI to react with the CNC hydroxyls in a comparable rate to that of p-NCOs. Increasing the TDI/CNCs molar ratio had a positive impact on carbamation efficiency, as long as it happens on the CNC surface, because it increased the amount of p-NCO per hydroxyl group in the reaction mixture leaving the o-NCO a smaller chance to react. Molar ratios of more than 3 and/or temperatures of more than 45 °C triggered the reaction to progress also in the amorphous regions, in which TDI tended to react using both of its isocyanates due to the increased accessibility and reactivity of the hydroxyl groups there. Under most of the carbamation conditions, the crystalline structure of the CNCs was not significantly altered. Overall, 35 °C and a molar ratio of 3 were found optimum to obtain a maximum carbamation efficiency (DS_NCO_/DS_TDI_) of 93%.

## Figures and Tables

**Table 1:** The carbamation conditions and the corresponding amounts of reactants, catalyst, and solvent. Time and CNC amount were kept constant (24 h and 1.0 g, respectively).

T (°C)	TDI/CNCs Ratio	Mass TDI (g)	Volume TEA (mL)	Volume Toluene (mL)
35	1	1.1	1.0	50.0
	2	2.2	2.0	48.2
	3	3.3	3.0	46.3
	4	4.4	4.0	44.4
	5	5.5	5.0	42.5
45	1	1.1	1.0	50.0
	2	2.2	2.0	48.2
	3	3.3	3.0	46.3
	4	4.4	4.0	44.4
	5	5.5	5.0	42.5
55	1	1.1	1.0	50.0
	2	2.2	2.0	48.2
	3	3.3	3.0	46.3
	4	4.4	4.0	44.4
65	1	1.1	1.0	50.0
75	1	1.1	1.0	50.0

**Table 2:** The impact of reaction conditions on degree of substitution of isocyanates (DS_NCO_), degree of carbamation (DS_TDI_), and the efficiency of CNC carbamation. Time and CNC amount were kept constant (24 h and 1.0 g, respectively).

T (°C)	TDI/CNCs Ratio	DS_NCO_ (%)	DS_TDI_ (%)	DS_NCO_/DS_TDI_ (%)
**35**	1	3.2 ± 0.0	4.5 ± 0.1	72
	2	5.5 ± 0.3	6.8 ± 0.2	81
	3	7.2 ± 0.3	7.7 ± 0.1	93
	4	7.2 ± 0.3	10.8 ± 0.8	67
	5	7.2 ± 0.3	17.4 ± 2.0	43
**45**	1	4.9 ± 0.3	7.4 ± 0.2	66
	2	5.8 ± 0.0	7.9 ± 0.2	73
	3	6.7 ± 0.3	8.4 ± 0.0	80
	4	7.2 ± 0.3	12.3 ± 2.0	58
	5	7.2 ± 0.3	21.2 ± 0.9	34
**55**	1	5.8 ± 0.6	9.6 ± 0.2	61
	2	6.4 ± 0.0	11.3 ± 0.3	56
	3	6.7 ± 0.3	12.5 ± 0.5	53
	4	7.2 ± 0.3	17.5 ± 1.1	41
**65**	1	5.5 ± 0.3	12.3 ± 0.1	45
**75**	1	4.9 ± 0.3	14.2 ± 0.3	34

## Supplementary Materials

The following are available online at https://www.mdpi.com/2073-4360/11/7/1164/s1, Figure S1: The estimation of DS_TDI_ for the carbamated CNCs using the N/C molar ratio obtained from EDX.
