# Supplementary material for: On the Para/Ortho Reactivity of Isocyanate Groups during the Carbamation of Cellulose Nanocrystals Using 2,4-Toluene Diisocyanate
Source: Polymers (Basel). 2019 Jul 8;11(7):1164. doi: 10.3390/polym11071164 (PMC6680866; doi:10.3390/polym11071164)
Supplement: Supplementary file 1 [file polymers-11-01164-s001.pdf]

Supplementary Materials

Determination of Carbamation Degree (DS_{TDI}) using EDX

The elemental composition of the original CNCs (DS_{TDI}=0%) was determined using EDX and based on it, the elemental composition of proposed carbamated samples of DS_{TDI} up to 33% was calculated to construct **Figure S1**. As a result, the DS_{TDI} for any carbamated CNC sample can be calculated using the equation in the figure after its N/C molar ratio is determined using EDX (**Figure S1**).

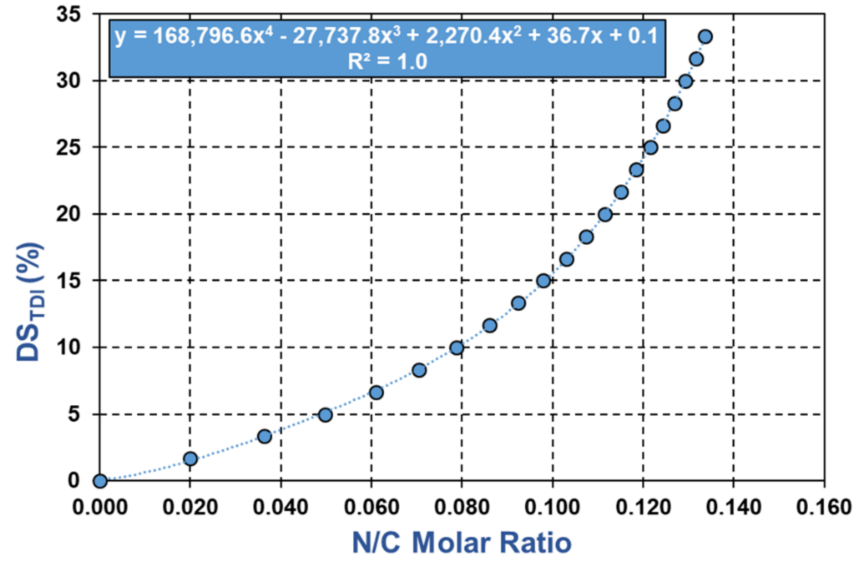

Figure S1. The estimation of DS_{TDI} for the carbamated CNCs using the N/C molar ratio obtained from EDX.

Determination of the Degree of Substitution of Free Isocyanates (DS_{NCO})

100 mL of DMSO were transferred to a 200 mL beaker before being acidified to a pH of 2.47±0.01 using 2 mL of 2 M HCl. The carbamated CNCs, after washed with DMSO, were transferred to the beaker. The pH started to increase because of isocyanate hydrolysis to amine groups. The hydrolysis was complete when no further increase in pH was observed (a maximum of 1 hour). All pH measurements were recorded at a temperature of 25 ± 0.5 ⁰C using a SevenEasy S20-KS pH meter equipped with InLab® Routine Pro electrode (Mettler Toledo, Giessen, Germany). DS_{NCO} was calculated based on the final pH value upon hydrolysis:

$$DS_{NCO} = \frac{mmol\ NCO}{mmol\ CNC\ Hydroxyls} = \frac{-24.3pH^4 + 290.3pH^3 - 1302.0pH^2 + 2598.7pH - 1946.0}{3 * (mass\ CNCs/162)} * 100\%$$
